# DWI-Based Neural Fingerprinting Technology: A Preliminary Study on Stroke Analysis

**DOI:** 10.1155/2014/725052

**Published:** 2014-08-12

**Authors:** Chenfei Ye, Heather Ting Ma, Jun Wu, Pengfei Yang, Xuhui Chen, Zhengyi Yang, Jingbo Ma

**Affiliations:** ^1^Department of Electronic and Information Engineering, Harbin Institute of Technology Shenzhen Graduate School, HIT Campus, University Town, Room 205C, C Building, Xili, Nanshan, Shenzhen 518055, China; ^2^Department of Neurology, Peking University Shenzhen Hospital, Shenzhen 18036, China; ^3^School of Information Technology and Electrical Engineering, The University of Queensland, St. Lucia, QLD 4072, Australia

## Abstract

Stroke is a common neural disorder in neurology clinics. Magnetic resonance imaging (MRI) has become an important tool to assess the neural physiological changes under stroke, such as diffusion weighted imaging (DWI) and diffusion tensor imaging (DTI). Quantitative analysis of MRI images would help medical doctors to localize the stroke area in the diagnosis in terms of structural information and physiological characterization. However, current quantitative approaches can only provide localization of the disorder rather than measure physiological variation of subtypes of ischemic stroke. In the current study, we hypothesize that each kind of neural disorder would have its unique physiological characteristics, which could be reflected by DWI images on different gradients. Based on this hypothesis, a DWI-based neural fingerprinting technology was proposed to classify subtypes of ischemic stroke. The neural fingerprint was constructed by the signal intensity of the region of interest (ROI) on the DWI images under different gradients. The fingerprint derived from the manually drawn ROI could classify the subtypes with accuracy 100%. However, the classification accuracy was worse when using semiautomatic and automatic method in ROI segmentation. The preliminary results showed promising potential of DWI-based neural fingerprinting technology in stroke subtype classification. Further studies will be carried out for enhancing the fingerprinting accuracy and its application in other clinical practices.

## 1. Introduction

Magnetic resonance imaging (MRI) has been widely employed in research as well as in clinical practice. For instance, diffusion weighted imaging (DWI) and diffusion tensor imaging (DTI) technologies provide remarkable detailed information of nervous system and have become the important examinations for neural diseases diagnosis in neurology department of hospital. Specifically, DTI measures the water diffusion situation in neural fibre so that it is frequently used to investigate the abnormal diffusion in the brain. Based on DWI principles, DTI can provide the contrast of the diffusion anisotropy that was further developed to trace the fibre tracts [1]. Both DWI and DTI technologies produce special contrast of nervous system in terms of diffusion ability, fibre integrity, fibre bundle directions, and so forth. In order to take advantage of these neuroimaging approaches, quantitative analysis is crucial for the image interpretation, which is also important for clinical applications. Quantitative measures, such as mean diffusivity (MD) and fractional anisotropy (FA), were proposed to measure the cellular diffusion state and the anisotropy of fibre tract in white matter [2] based on DTI. An increasing number of quantitative methods were introduced to DTI data analysis, such as voxel-based analysis (VBA) [3] and tract-based spatial statistics (TBSS) [4, 5]. These methods can automatically localize the lesion in the brain by comparing patients' images with a normal control group [6]. However, the existing quantitative analysis methods of DTI are sensitive to the lesion location but not the physiological changes in nature. For instance, a lesion in the brain can be localized by VBA according to the FA value changes while the inherent physical meaning for such changes cannot be reflected by this analysis. According to the imaging principles, images of DTI and DWI can not only provide structural information but also contain physiological meanings [7]. Further development on the quantitative analysis will facilitate interpretation of DWI data that is much helpful in both neuroscience research and clinical practice.

Stroke is a common neural disease especially for the elderly and the people with hypertension [8]. In clinical applications, DWI has shown accurate identification of ischemic tissue and the ability to discriminate between dead and salvageable ischemic brain [9–11]. Acute ischemic lesions in DWI can be detected with greater sensitivity than conventional MRI, such as T1 and T2 weighted imaging [12–15]. Besides, defining different stroke states by MRI images is important to follow up patients' response to a therapy. It is reported that decreased apparent diffusion coefficient (ADC) values indicate good sensitivity and specificity in an infarct less than 10 days old [16]. Appearances on DWI images following stroke also vary in different states [17]. However, the performance of acute infarct detection or stroke state determination is unsatisfactory by simply identifying hyperintensity or hypointensity on DWI images by thresholding [18]. Current quantitative measures, such as ADC and FA [19], are employed to provide different contrasts of lesion to identify the infarction area and location. Better utility of DWI and DTI data would make it possible to identify subtypes of stroke, which will enhance the diagnosis of physiological variation of the patients and thus affect further clinical management. A quantitative measure, which contains comprehensive features of the nerve, should be developed to exploit the rich information in DWI images for detecting subtypes of ischemic stroke.

In this study, we proposed a method called DWI-based neural fingerprinting to characterize the neural physiological changes that can be used for subtype classification of ischemic stroke. The “fingerprinting” concept was borrowed from magnetic resonance fingerprinting (MRF) [20] technique, permitting the accelerating acquisition of multiple magnetic resonance parameters, while in current proposal the “fingerprint” refers to a feature vector constructed from the DWI images with different diffusion gradients that contains comprehensive neural information. As anisotropy measurement shows sensitivity to degrees of fibre damage in disease affecting white matter [21–23], we hypothesize that specific diffusivity change within ischemic stroke could be considered as a fingerprint reflecting unique neural property. Therefore, pathological changes within the infarction of patients after stroke would be associated with the fingerprint extracted from the DWI data with different gradients. By applying clustering algorithms on the fingerprints, the subjects can possibly be classified into normal controls, patients with acute stroke, and patients with stroke sequela.

## 2. Methodology

### 2.1. Data Acquisition and Image Preprocessing

The present study adopted retrospective clinical data from the Neurology Department of Peking University Shenzhen Hospital. Clinical data from 19 subjects (13 men and 6 women, 49 ± 19 years old) were collected, where MRI examinations with the same protocol were conducted on the subjects. The 19 subjects were diagnosed as eight healthy people (4 men and 4 women, 31 ± 4 years old), eight with acute stroke lesions (6 men and 2 women, 64 ± 15 years old), and three with stroke sequela (3 men, 58 ± 8 years old). For the 11 patients with acute stroke and stroke sequela, 11, 10, and 9 lesions were located in the internal capsule, the striatocapsular, and the motor cortex, respectively. Extensive information about participants' health status has been obtained through symptomatic evaluation. The diagnosis reports were issued by two neurologists in the Peking University Shenzhen Hospital. All the participants underwent MR imaging with 1.5 T Siemens Sigma System (Siemens Medical Systems). The typical MRI protocol consisted of turbo spin echo (TSE) sequence to generate T2 weighted images (TE = 89 ms; TR = 4000 ms; flip angle = 150°; acquisition matrix = 768 × 624; FOV = 230 × 187 mm^2^) and single-shot echo-planar spin-echo (EPSE) sequence to obtain DWI images (TE = 88 ms; TR = 2700 ms; flip angle = 90°; acquisition matrix = 128 × 128; FOV = 250 × 250 mm^2^; in-plane resolution 1 × 1 mm^2^;* b* = 1000 s/mm^2^; 20 diffusion weighted gradient directions and 1 without diffusion weighting). Nineteen axial sections in 6.5 mm slice gap with 5 mm thickness were obtained.

The 20 DWI images in Digital Imaging and Communications in Medicine (DICOM) format of each subject were imported into the SPM8 software (Welcome Trust Centre, UCL) for preprocessing [24], involving spatial normalization to the standard MNI space [25, 26] and Gaussian smoothing (FWHM of 3 mm) [27].

### 2.2. ROI Segmentation and Fingerprint Construction

Obtaining diffusion weighted signals of the infarct is much dependent on the accurate localization of infarct area. Three methods were used in the present study, named manual, semiautomatic, and automatic ROI segmentation. The manual ROI was segmented by clinicians, which was also supposed to be the reference for the semiautomatic method.

**Figure 1:**
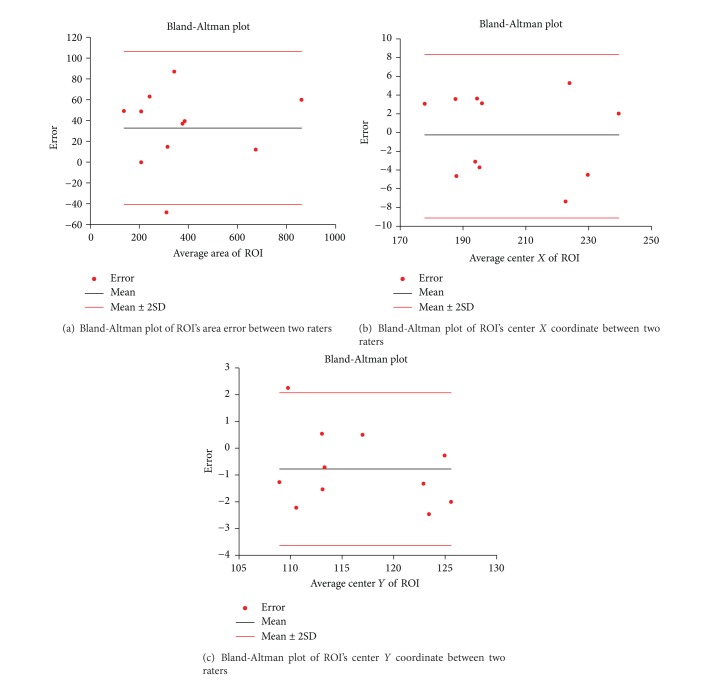
Bland-Altman plot of ROI between two raters.

**Figure 2:**
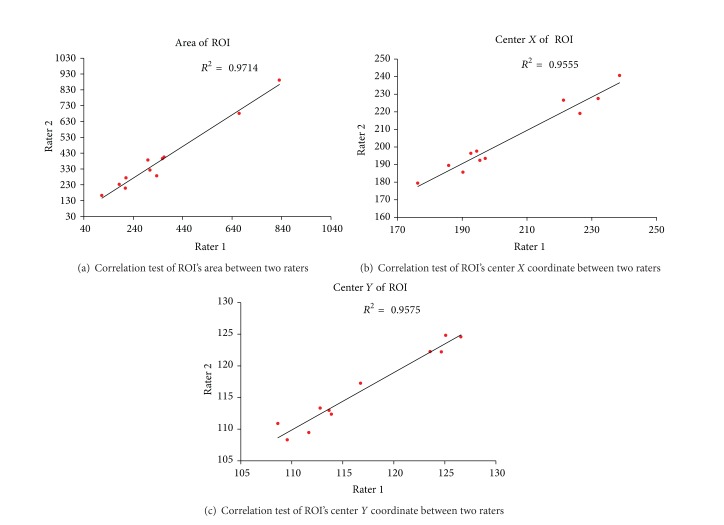
Correlation test of ROI between two raters.

**Figure 3:**
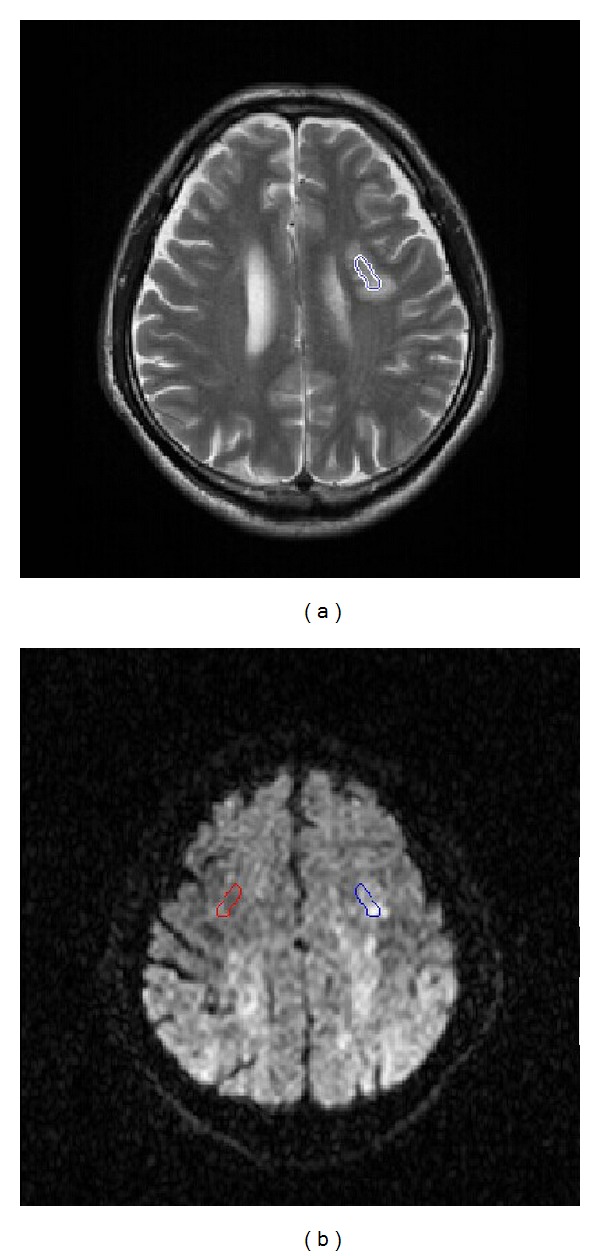
Manual drawings of ROIs of stroke lesion on DWI images. The stroke lesion was observed in the brain DWI image. (a) T2 weighted image facilitated infarct location on the corresponding DWI images. (b) Stroke lesion was localized in DWI image (blue circle) by image registration with T2 image, while the contralateral region generated automatically in red.

First, the manual ROI was defined by clinicians. For stroke patients, two experienced neurologists blinded to clinical symptoms drew target ROI of stroke lesion independently on T2 weighted images while taking the T1 and ADC images as reference. To evaluate the drawing agreement of different operators, the error of ROI's areas and center coordinates regarding each participant were evaluated by Bland-Altman plots and correlation coefficients. The interrater reliability of ROI is shown in Figures 1 and 2. All evaluation measures show good agreement between the two operators (correlation coefficients > 0.95) to guarantee robust and accurate ROI segmentation. Then, the intersection parts of ROIs were mapped to the corresponding DWI images to produce the final infarct location through multimodal registration, as shown in Figure 3. For normal subjects, one arbitrary cerebral hemisphere was selected as target ROI to be investigated as there was no infarct in their brain. The manual ROI was supposed to provide the most accurate segmentation on the stroke lesion due to the professional knowledge of the operators.

**Figure 4:**
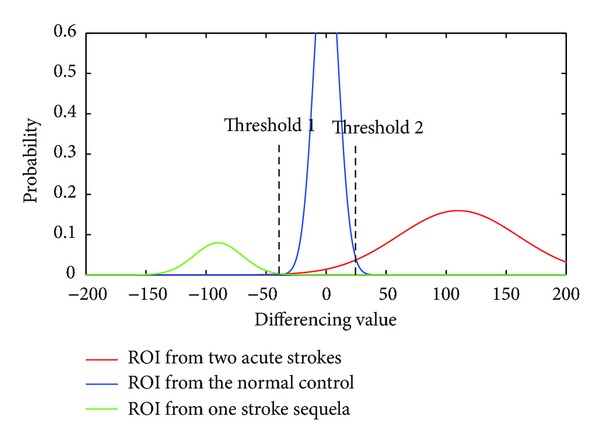
The probability density curve of voxelwise differencing value. Blue curve represents the normal control; red curve represents the acute stroke group; green curve represents the stroke sequela group. Two thresholds are determined to discriminate the different distributions.

A semiautomatic method for ROI location was proposed based on morphological segmentation. Firstly, DWI images of each participant were registered to a normal brain template. Then, we averaged 20 slices of registered DWI images into one image for each subject. Three stroke subjects were arbitrarily selected from normal subjects, patients with acute stroke, and patients with stroke sequela, respectively. The ROIs were drawn accordingly to provide necessary references to differentiate normal brain tissue and stroke lesions. Image signal intensity was compared voxelwise between the ROI and the mirror area on the opposite hemisphere for each subject. The histogram of the signal intensity difference was mapped to derive the optimal thresholds to differentiate the lesion from the normal brain tissue.  Figure 4 shows the probability density function based on the histogram, where two optimized thresholds were determined as the criteria for lesion identification. Then, the two thresholds were used for lesion ROI segmentation on other patients' DWI images.

**Figure 5:**
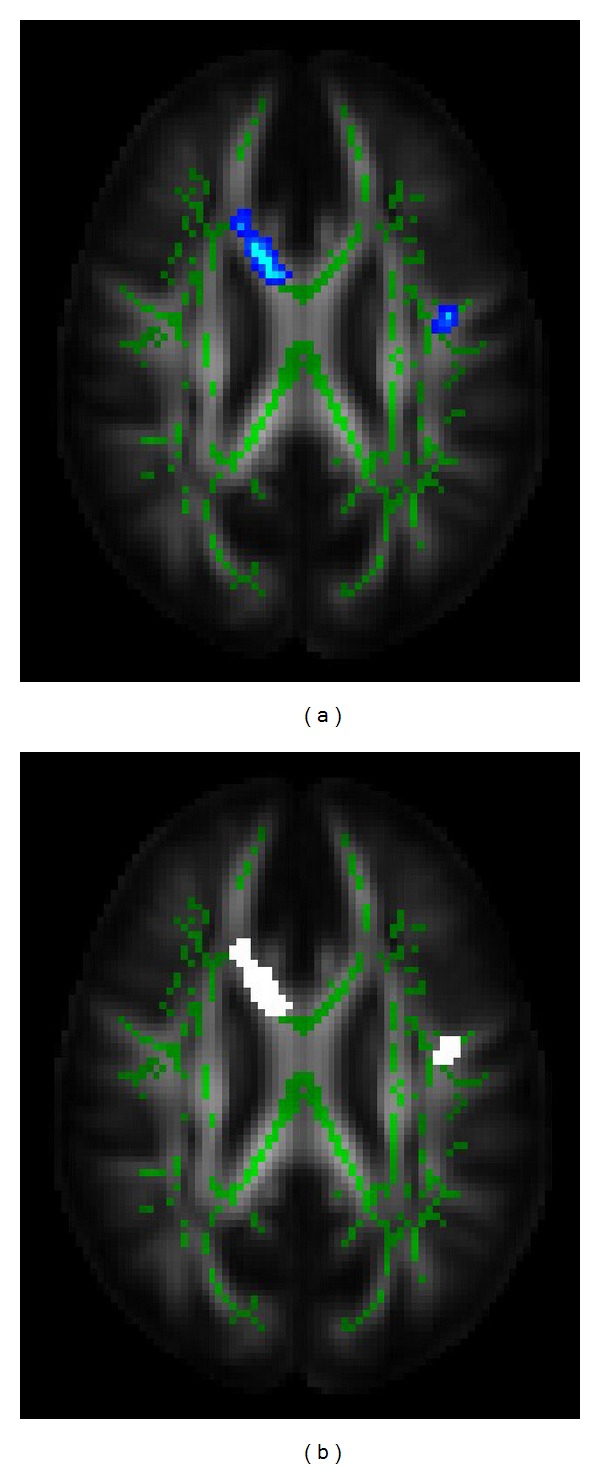
Automatic ROI generation on FA map by TBSS. (a) Blue regions indicate significantly decreased FA (*P* < 0.005) in patients with stroke relative to normal controls. (b) White regions indicate the corresponding lesion ROIs. Green regions represented the mean FA skeleton.

In order to segment the lesion ROI automatically, we also proposed a TBSS based method. We hypothesized that the stroke lesion in the brain would contain water diffusibility changes that vary the fractional anisotropy of the pixel. Then, through TBSS approach, pixels with significant FA change were detected and used to compose the lesion ROI. This method was implemented using FMRIB Software Library (FSL 4.1.9; http://www.fmrib.ox.ac.uk/fsl) [28]. First of all, a brain template in the software was identified as a common registration target. We aligned all subjects' FA images to this target by nonlinear registration. Then, a skeletonised mean FA image was created by a nonmaximum suppression perpendicular to the local tract structure. Each subject's FA image (aligned) was projected onto the skeleton by filling the skeleton with FA values from the nearest relevant tract center. Finally, based on the voxelwise statistics across the stroke patients and the normal controls, the target ROI was defined as the voxels with significant difference (uncorrected *P* < 0.005), as illustrated in Figure 5.

**Figure 6:**
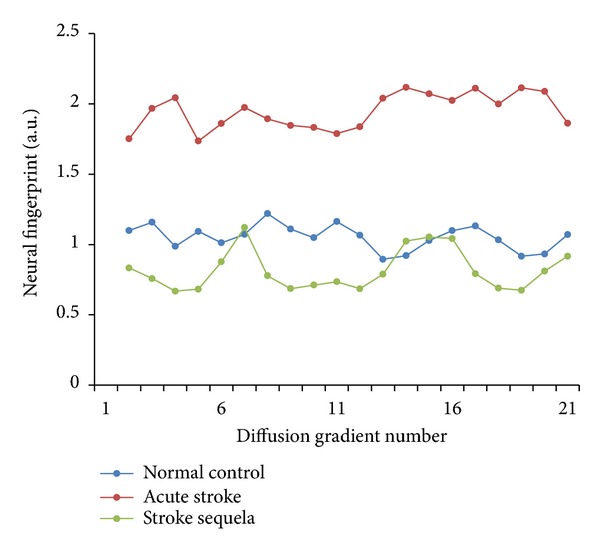
An example of neural fingerprints from manual ROI in DWI images. These neural fingerprints are averaged from manual ROI method. Blue curve represents the normal control; red curve represents the acute stroke group; green curve represents the stroke sequela group.

After segmenting the target ROIs with the above three methods, neural fingerprints can be constructed from them, respectively, to reduce intersubject variations in DWI intensity, the mirror ROI (the contralateral region) corresponding to the target ROI was used as a reference. An example of mirror ROI from the manual segmentation is shown by the red circle generated automatically in Figure 3. The design of diffusion gradients is also critical for the construction of neural fingerprints. Considering that the arrangement of diffusion gradients in the three-dimensional space did not affect classification of neural fingerprints only if applying a constant order, we applied 20 diffusion gradients distributed randomly in a constant order in every subject's DWI data. The neural fingerprint for each ROI segmentation method is defined as a vector of 20 elements. Each element is a ratio between the mean image intensities within the target ROI and the mirror ROI calculated with each diffusion gradient. An example of neural fingerprint from manual ROI in DWI images is shown in Figure 6.

### 2.3. Clustering

To validate the DWI-based neural fingerprinting method, unsupervised learning was employed to cluster the nineteen subjects. Fingerprints used are the ratio values calculated from manual, semiautomatic, and automatic ROIs, respectively. As the clustering metrics, two types of distance were employed [29]. The Euclidean distance was calculated as follows:

(1) $$\begin{array}{c} D_{E}=\sqrt{\left(x_{s}-x_{t}\right){\left(x_{s}-x_{t}\right)}^{\prime }}, \end{array}$$

where **x**_**s**_ and **x**_**t**_ are feature vectors of two subjects (the principal components of average image signal intensity sequences). The Cosine distance was calculated as follows:

(2) $$\begin{array}{c} D_{C}=1-\frac{x_{s}x_{t}^{\prime }}{\sqrt{\left(x_{s}x_{s}^{\prime }\right)\left(x_{t}x_{t}^{\prime }\right)}}. \end{array}$$

The* K*-means algorithm [30, 31] was performed for fingerprint clustering. By setting the number of clusters* k*, each observation was assigned by minimizing the least within-cluster sum of squares (WCSS) until the assignments no longer change. WCSS was defined as follows:

(3) $$\begin{array}{c} \underset{s}{\arg\;\min}\overset{k}{\underset{i=1}{\sum }}\;\overset{}{\underset{x_{j}\in S_{i}}{\sum }}{||x_{j}-\mu_{i}||}^{2}, \end{array}$$

where *x*_*j*_ belongs to observation (*x*_1_, *x*_2_,…, *x*_*n*_), **S** = (*S*_1_, *S*_2_,…, *S*_*k*_) is* k* clusters, and *μ*_*i*_ is the mean of points in *S*_*i*_. We compared 6 sets of clustering results obtained using the combinations of the three ROI methods and the two distance metrics (Euclidean and Cosine distance), respectively.

To evaluate the clustering results,* F* score, a common metric to estimate how close the clustering is to the predetermined benchmark classes, was calculated as follows [32]:

(4) $$\begin{array}{c} F=\overset{s}{\underset{j=1}{\sum }}\frac{\left|P_{j}\right|}{\sum_{i=1}^{s}\left|P_{i}\right|}\cdot \underset{1\leq i\leq m}{\max}\frac{2\cdot P\left(P_{j},C_{i}\right)\cdot R\left(P_{j},C_{i}\right)}{P\left(P_{j},C_{i}\right)+R\left(P_{j},C_{i}\right)}, \end{array}$$

where *P* is the preclassified sample clusters and *s* is its corresponding number of clusters. *C* is the sample clusters and *m* is its corresponding number of clusters. Precision *P*(*P*_*j*_, *C*_*i*_) is the correct results divided by the number of all returned results, and recall *R*(*P*_*j*_, *C*_*i*_) is the number of correct results divided by the results that should have been returned [33]. The* F* score can be interpreted as a weighted average of the precision and recall, where* F* score reaches its best score at 1 and worst score at 0.

## 3. Results

Based on the fingerprint, the clustering results are shown in Table 1. The clinical diagnosis in the first column is taken as the standard reference, where “1” represents the normal control, “2” represents the group with acute stroke lesions, and “3” represents the group with stroke sequela. For the manual ROI method, it can be observed that clustering result approached 100% accuracy with Euclidean distance. In other words, the fingerprint based on the manual ROI with Euclidean distance gives the best clustering performance compared to others. However, the accuracy is relatively poor when applying Cosine distance (accuracy = 68%, 95% CI: 48%–89%). For the semiautomatic segmentation method, two subjects with acute stroke (Subjects 13 and 15) were falsely included into the normal group with Cosine distance, and the corresponding accuracy remained at high level (accuracy = 89%, 95% CI: 76%–97%). It indicates that the semiautomatic method is only sensitive to stroke sequela. With Euclidean distance, the accuracy for semiautomatic method is 74% (95% CI: 54%–93%). For automatic TBSS method, mismatch occurs much more frequently in all of the three groups, as the accuracy with Euclidean and Cosine distance is 58% (95% CI: 36%–80%) and 63% (95% CI: 41%–85%), respectively.

**Figure 7:**
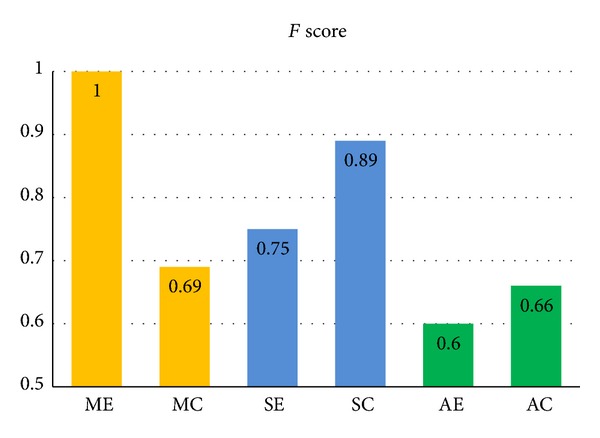
*F* scores using two distance metrics combined with manual, semiautomatic, and automatic ROIs. ME: Euclidean distance using manual ROI; MC: Cosine distance using manual ROI; SE: Euclidean distance using semiautomatic ROI; SC: Cosine distance using semiautomatic ROI; AE: Euclidean distance using automatic ROI; AC: Cosine distance using automatic ROI.

The comparison between* F* scores provides an overall clustering evaluation for each method, as shown in Figure 7.* F* score of the manual ROI method with Euclidean distance shows accurate clustering performance (*F* = 1). The difference of* F* scores between two distances for manual method is relatively larger than that in other methods. For the semiautomatic segmentation method, the* F* scores are higher (i.e., 0.75 and 0.89) than those in automatic TBSS method (i.e., 0.6 and 0.66) with both distances. In other words, the clustering of semiautomatic method provided a better classification result than that of automatic TBSS method.

## 4. Discussions

With the development of neuroimaging technology, the detection and analysis of ischemic stroke relying on MRI have achieved high reliability and availability. T1, T2, DWI, DTI, and ADC maps are commonly used in clinics to detect stroke lesions based on hyperintensity or hypointensity on the images. Medical doctors usually check several types of MRI images to determine the subtypes of the ischemic stroke lesion. The DWI-based neural fingerprinting method presented here is a new quantitative approach, which takes advantage of diffusion weighted data to construct a feature vector representing the unique neurophysiological information of the brain tissue. We further applied the fingerprint to determine the subtypes of ischemic stroke which can be only based on the DWI images on different diffusion gradients. The fingerprint produced in this study can quantitatively measure pathological change of neural tissue, which reflects the specific states of stroke lesions, as shown in Figure 6. The preliminary results basically validated the hypothesis that neural physiological change in ischemic stroke can be reflected by the diffusion signal variation on different gradients. Further, the fingerprint proposed in current study can be used for subtype determination for ischemic stroke. Based on neural fingerprint technology, it is possible to further develop a tool to assist medical doctors in the diagnosis of stroke disease.

For the fingerprint generation, lesion ROI segmentation is a key step for the final clustering results. It appeared that the manual ROI method yielded the best clustering result among the three segmentation methods. As shown in Table 1, a perfect match between clustering results and clinical reference occurs when manual ROI with Euclidean distance was used (*F* score = 1). It indicates that different phases of ischemic stroke could be distinguished accurately when lesions have been perfectly localized and distance has been properly defined. It also implies that DTI protocol is suitable to generate fingerprints for ischemic stroke. The precise mechanism leading to diffusion changes of ischemic stroke is still not for certain. Wallerian degeneration in the nervous system was found in animal model [34], which involved the breakdown of the myelin sheath and disintegration of axonal microfilaments [35]. Although disruption of myelin and axons around acute infarct lesions might be expected to increase the water diffusivity, an accumulation of cellular debris from the breakdown of axons may hinder water molecule motion [36], which is more likely to occur in late stage of stroke. Another explanation could be the redistribution of extracellular water into the intracellular compartment, which leads to the shrinkage of extracellular space overtime [37]. These scientific findings may explain why the fingerprint constructed by the DTI images can distinguish the subtypes of the ischemic stroke.

The semiautomatic and automatic ROI segmentation methods were proposed to develop automatic neural fingerprint construction method. The* F* scores for both methods are lower than those obtained in manual ROI with Euclidean distance. It is probably due to the inaccuracy in the ROI localization of stroke lesions. For the semiautomatic method, the thresholds were determined only by three subjects' data, which may not reflect the comprehensive features of the lesion. More samples in the training set are necessary to improve a priori knowledge of the lesion feature. As for the automatic method, the inherent hypothesis, FA value varied significantly for stroke, may not be perfect for the ischemic stroke lesion. In addition, FA images without diffusion tensor orientation information could contribute to false location of significantly abnormal areas. Another limitation may come from the TBSS itself. Usually TBSS method is based on the group comparison, not for single brain lesion detection. The weighting of one specific lesion from a single patient is weakened by the group statistical analysis. The semiautomatic segmentation method overcomes the constraints of TBSS; thus, it has a better discrimination as shown in the clustering result. But the subjects with acute stroke and the normal control fail to be classified perfectly, probably because that threshold 1 with mixed area under curve is more difficult to determine than threshold 2 (Figure 4). The clustering result also implies that appropriate distance metrics should be used to achieve sufficient discriminative power of DWI-based neural fingerprinting. The investigation of optimal distance metric is one of the future works.

The current study is the first effort trying to construct a fingerprint representing neurophysiological information and we implemented the technology on ischemic stroke classification. As an ongoing research, a system should be finally built up to achieve the true fingerprinting function, that is, identification. The identity here refers to the neural specific physiology, which can be represented by a unique fingerprint. In the current study, we chose to use the DWI images on different diffusion gradients to construct the fingerprint. Other MRI protocols are also possible for fingerprint construction once the protocol can provide unique pattern of specified neural physiology. Huge efforts should be carried out to construct a fingerprint bank, which covers massive amounts of neural fingerprints with matched information (age, gender, pathology, etc.). Then, the neural fingerprinting technology can be finally realized by providing neural fingerprint identification or verification through comparing an arbitrary neural fingerprint that comes from a patient to the fingerprint bank. Many applications can be rooted from the neural fingerprinting technology, such as autodiagnosis and risk evaluation for some diseases. Despite the specific brain disease detection, the quantification of general neural property distributed in the whole nervous system relies on the integrated fingerprint bank. Building up the whole system would involve the professional knowledge and efforts from the medical doctors, biologists, engineers, MRI physicists, and so forth.

In conclusion, this preliminary study demonstrated that the proposed DWI-based neural fingerprinting method had the potential to classify brain abnormalities, such as acute stroke and stroke sequela, due to its ability to exploit comprehensive information contained in DWI data. Further development on such technology could assist clinical practice in the future.

## Figures and Tables

**Table 1:** Clustering result and evaluation.

	Clinical reference	Manual ROI		Semiautomatic ROI		Automatic ROI	
		Euclidean distance	Cosine distance	Euclidean distance	Cosine distance	Euclidean distance	Cosine distance
Subject 1	1	1	1	1	1	1	1
Subject 2	1	1	1	1	1	1	1
Subject 3	1	1	1	1	1	1	1
Subject 4	1	1	1	1	1	1	3
Subject 5	1	1	1	1	1	1	1
Subject 6	1	1	1	1	1	1	1
Subject 7	1	1	1	1	1	3	1
Subject 8	1	1	1	1	1	3	1
Subject 9	2	2	1	3	2	1	3
Subject 10	2	2	2	3	2	2	3
Subject 11	2	2	1	2	2	2	2
Subject 12	3	3	3	1	3	3	3
Subject 13	2	2	1	2	1	1	3
Subject 14	2	2	1	2	2	2	2
Subject 15	2	2	2	2	1	2	2
Subject 16	3	3	3	1	3	2	2
Subject 17	2	2	1	3	2	3	1
Subject 18	2	2	1	2	2	3	3
Subject 19	3	3	3	1	3	1	3
*F* value		1	0.69	0.75	0.89	0.6	0.66

Clinical reference classification and DWI-based neural fingerprinting clustering results are shown. For the clinical reference, 1 represents the normal control; 2 represents the group with acute stroke lesion; and 3 represents the group with stroke sequela. For manual, semiautomatic, and automatic ROI methods, different numbers are different clustering labels.
